# Long non-coding RNA *MIR4300HG* polymorphisms are associated with postoperative nausea and vomiting: a genome-wide association study

**DOI:** 10.1186/s40246-020-00282-4

**Published:** 2020-09-14

**Authors:** Shigekazu Sugino, Daisuke Konno, Yosuke Kawai, Masao Nagasaki, Yasuhiro Endo, Tomo Hayase, Misako Yamazaki-Higuchi, Yukihiro Kumeta, Shunsuke Tachibana, Katsuhiko Saito, Jun Suzuki, Kanta Kido, Nahoko Kurosawa, Akiyoshi Namiki, Masanori Yamauchi

**Affiliations:** 1 Department of Anesthesiology and Perioperative Medicine, Tohoku University School of Medicine, Seiryo-machi 2-1, Sendai, Miyagi 980-8575 Japan; 2 Department of Anesthesia, Otaru General Hospital, Wakamatsu 1, Otaru, Hokakido 047-8550 Japan; 3 Department of Integrative Genomics, Tohoku University Tohoku Medical Megabank Organization, Seiryo-machi 2-1, Sendai, Miyagi 980-8575 Japan; 4 Department of Pharmacy, Hokkaido Pharmaceutical University School of Pharmacy, Maeda 7-15, Sapporo, Hokakido 006-8590 Japan; 5 Department of Anesthesiology, Kanagawa Dental University Graduate School of Dentistry, Inaoka 82, Yokosuka, Kanagawa 238-8580 Japan; 6 Hospital Administrator, Otaru General Hospital, Wakamatsu 1, Otaru, Hokakido 047-8550 Japan

**Keywords:** Long non-coding RNA, Single-nucleotide polymorphism, Postoperative nausea and vomiting

## Abstract

**Background:**

Genetic factors such as single-nucleotide polymorphisms (SNPs) play a key role in the development of postoperative nausea and vomiting (PONV). However, previous findings are not widely applicable to different populations because of population-specific genetic variation. We developed a Japanese-specific DNA microarray for high-throughput genotyping. The aim of the current study was to identify SNPs associated with PONV on a genome-wide scale using this microarray in a sample of Japanese surgical patients.

**Methods:**

Associations between 659,636 SNPs and the incidence of PONV 24 h after surgery in a limited sample of 24 female patients were assessed using the microarray. After imputation of genotypes at 24,330,529 SNPs, 78 SNPs were found to be associated with the incidence of PONV. We chose 4 of the 78 SNPs to focus on by in silico functional annotation. Finally, we genotyped these 4 candidate SNPs in 255 patients using real-time PCR to verify association with the incidence of PONV.

**Results:**

The T > C variant of rs11232965 in the long non-coding RNA MIR4300HG was significantly associated with reduced incidence of PONV among genotypes and between alleles (*p* = 0.01 and 0.007).

**Conclusions:**

We identified a novel SNP (rs11232965) in the long non-coding RNA MIR4300HG that is associated with PONV. The rs11232965-SNP variant (T > C) is protective against the incidence of PONV.

**Trial registration:**

This study was registered at the UMIN Clinical Trials Registry (Identifier: UMIN000022903, date of registration: June 27, 2016, retrospectively registered.

## Background

Postoperative nausea and vomiting (PONV) is the most unpleasant complication of surgical recovery worldwide [1]. It occurs after emergence from general anesthesia and frequently produces individual differences in patient discomfort and dissatisfaction because PONV incidence differs significantly among patients. Approximately 30% of all patients experience PONV, and in a subpopulation of high-risk patients, incidence increases to 80% [1]. Previous large-scale clinical trials identified postoperative opioids, inhalation anesthetics, nitrous oxide, long surgery duration, female sex, non-smoking status, and a history of motion sickness or PONV during previous surgeries as risk factors [2]. However, these clinical factors cannot completely predict the PONV incidence in individual patients.

Genetic factors like single-nucleotide polymorphisms (SNPs) contribute to PONV [3, 4]. Several SNPs in candidate genes (e.g., *HTR3A*, *HTR3B*, *CYP2D6*, *TACR1*, *DRD2*, *COMT*, and *OPRM1*) have been examined for associations with PONV in surgical patients [3]. Although the most frequently studied SNP in *OPRM1* (rs1799971, also known as A118G) might be associated with PONV, it remains controversial [5, 6]. In other PONV-associated genes, the effect of SNPs on PONV was smaller than that of clinical factors [7, 8]. We hypothesize unknown genetic variation at genes besides these candidates is critically involved in PONV regulation. Additional genotyping and association tests are needed at the genome-wide level.

Rs2165870-SNP in *CHRM3*, which encodes the muscarinic acetylcholine receptor, was recently identified as a predictor for PONV by a genome-wide association study in a Caucasian population [9, 10]. These two studies are the only genome-wide association studies revealing significant SNPs associated with PONV. This finding is not widely applicable because genetic variation is affected by race and ethnicity [11, 12]. A genome-wide association study in each genetically homogenous population is a prerequisite for modern human genomic research.

We conducted a genome cohort study as part of the Tohoku Medical Megabank Project. We constructed the reference panel (1KJPN) of SNPs based on complete genome sequencing of 1070 Japanese individuals [13, 14]. We designed and developed a Japanese-specific DNA microarray based on the 1KJPN data for high-throughput SNP genotyping. This array provides excellent whole-genome coverage for SNPs in the Japanese population and is better than any commercially available SNP array. In addition, our array is suitable for whole-genome imputation of Japanese individuals [15, 16]. The aim of this study is to determine SNPs associated with PONV on a genome-wide scale using this DNA microarray in Japanese surgical patients.

## Materials and methods

### Anesthetic procedures in the two cohorts

This study was registered at the UMIN Clinical Trials Registry (Identifier: UMIN000022903, principal investigator: Shigekazu Sugino, date of registration: June 27, 2016, retrospectively registered, https://upload.umin.ac.jp/cgi-open-bin/ctr_e/ctr_view.cgi?recptno=R000026392). After registration, 264 Japanese adults (20–85 years of age) with ASA physical status of 1 or 2 were enrolled in this study. This manuscript adhered to applicable STREGA guidelines [17].

In 179 patients, epidural catheters were inserted through the T9–L2 interspaces before anesthetic induction. General anesthesia was maintained with 0.5–2.0% sevoflurane. Intraoperative use of nitrous oxide was at the discretion of the attending anesthesiologist. Three to five milliliters of 1.5% lidocaine and 5 μg/mL of epinephrine were administered epidurally at least every 30 min. At the end of the surgery, an epidural analgesia was administered as a continuous 4-mL/h infusion of ropivacaine (0.2%) and fentanyl (2 μg/mL) for postoperative pain control (Epi cohort).

In the other 85 patients, general anesthesia was maintained with 1.5–2.5% sevoflurane and 0.1–0.5 μg/kg/min remifentanil. Intraoperative use of nitrous oxide was at the discretion of the attending anesthesiologist. All patients received 25 μg bolus doses of intravenous (i.v.) fentanyl at 10-min intervals in the recovery room after surgery to control early postoperative pain. Intravenous patient-controlled analgesia (IV-PCA) was then initiated with a fentanyl solution of 0.4 μg/kg/min. A PCA pump (Coopdech Syrinjector PCA Set, DAIKEN Medical Co., Japan) was used with the following parameters: demand dose of 0.4 μg/kg of fentanyl, background infusion of 0.4 μg/kg/min, and lockout interval of 10 min (IV-PCA cohort).

### Assessment of PONV

Patient demographic information, including age, sex, height, weight, smoking status, history of motion sickness, and type and duration of surgery, was collected. In both cohorts, PONV was assessed 24 h postoperatively. PONV severity was assessed using a 4-point scale: 0 (no nausea), 1 (sensation of discomfort), 2 (severe nausea), and 3 (vomiting or retching). A PONV score of 2 or 3 was defined as an incidence of PONV. Only patients with a PONV score of 3 were treated with 10 mg of metoclopramide via i.v. Patients and anesthesiologists were blinded to the patient’s genotype throughout the postoperative assessment period and data analysis.

### Fentanyl blood concentration measurement

Ten milliliters of blood from each patient was collected 24 h postoperatively in heparinized tubes. Blood samples were immediately placed on ice, centrifuged, and stored at − 80 °C. Aliquots (270 μL) of serum samples were mixed with 30 μL internal solution (i.e., 0.1 μg/mL flurazepam), 700 μL n-hexane, and 300 μL ethylacetate. These mixtures were vortexed and centrifuged at 13,200 rpm for 10 min at room temperature. The organic phase was transferred to a sterile microtube and evaporated until dry. The residues were redissolved in 500 μL of mobile phase and transferred to an autosampler vial. An aliquot (10 μL) was injected into a high-performance liquid chromatograph (1200 series; Agilent Technologies, Santa Clara, CA, USA) and a tandem mass spectrometer (3200 QTrap; AB Sciex, Framingham, MA, USA). Separation was performed at 30 °C on a 2.5 mm × 150 mm, 5-μm C30 analytical column (Develosil RPAQUEOUS; Nomura Chemical, Aichi, Japan) coupled with a 1.5 mm × 10 mm guard column (RP-AR-S; Nomura Chemical). The mobile phase was prepared using a mixture of 50:50 formic acid (0.05%) to methanol (99%), and the flow rate was maintained at 0.2 mL/min. In the second quadrupole, fentanyl and flurazepam were monitored by the respective transitions of *m/z* 338.4 to 105.0 and *m/z* 389.2 to 316.2 with a collision energy of 51 eV. The standard curve was linear from 0.1 to 10 ng/mL.

### Genomic DNA extraction and quality control

Genomic DNA was extracted from 2 mL whole blood obtained from patients before induction of anesthesia using a Puregene Blood Core Kit B (Qiagen, Hilden, Germany). The quality and quantity of DNA were checked using a NanoDrop spectrophotometer (NanoDrop Technologies, Wilmington, DE, USA). Samples with an absorbance ratio lower than 1.6 at 260 and 280 nm were excluded from further analysis. The samples were stored at 4 °C until use.

### Genotyping of SNPs by Japanese-specific designed DNA microarray

Twenty-four patients were selected to explore SNPs associated with the incidence of PONV. Fourteen patients (female, undergoing gynecological open abdominal surgery, 7 of 14 patients had PONV) were collected from the Epi cohort, and ten patients (female, undergoing mastectomy, 5 of 10 patients had PONV) were collected from the IV-PCA cohort. Therefore, in the selected cohort, 12 patients experienced PONV, and the other 12 patients did not. This cohort was modeled as a clinically typical situation with frequent PONV occurrence.

Two hundred nanograms of genomic DNA from each individual was used to genotype 659,636 SNPs with a custom-made DNA microarray (Japonica Array; Toshiba Corporation, Tokyo, Japan). Genotype calling was conducted using the apt-probeset-genotype program in Affymetrix Power Tools (ver. 1.18.2; Thermo Fisher Scientific Inc., Waltham, MA, USA). All samples passed the recommended sample quality control metric (dish quality control > 0.82 and sample call rate > 97%). Quality control for SNPs was conducted using the Ps classification function in the SNPolisher package (version 1.5.2, Thermo Fisher Scientific Inc.). SNPs that were classified “recommended” by the Ps function were retained. SNPs with a call rate of less than 99.0%, significant deviation from Hardy–Weinberg equilibrium (HWE; *p* < 0.0001), or a minor allele frequency (MAF) of less than 0.5% were excluded from downstream analyses.

### Genotype imputation following genome-wide association study

Imputation of missing genotype data from the 24 patients was performed using IMPUTE2 (ver. 2.3.1) [18]. Pre-phasing was first conducted with these SNPs using SHAPEIT (v2.r644) with options --burn 10, --prune 10, and --main 25 [19]. Genotype imputation was performed on phased genotypes with IMPUTE2 using the 1KJPN panel. For IMPUTE2, the applied options were -Ne 2000, -k hap 1000, -k 120, -burnin 15, and -iter 50.

After genome-wide imputation with the Japonica array™, we cleaned the control sample data by applying quality control parameters of SNP call rate ≥ 95%, a MAF ≥ 1%, and HWE p ≥ 0.001. The 6,714,496 SNPs and insertions and deletions (indels) on autosomes that passed these quality control filters were used for the genome-wide association study. Associations between the PONV incidence and alleles of each SNP were analyzed by PLINK (ver. 1.9) [20]. A Manhattan plot was created to visualize genome-wide associations with qqman.

### Variant annotation and determination of candidate SNPs

SNPs with statistically significant associations with PONV incidence were ranked for variant annotation (*p* < 0.0001, see the “Results” section). High-confidence SNPs with a *p* value of less than 10^−4^ were annotated using the Variant Annotation Integrator of the UCSC Genome Browser [21]. After the annotation, SNPs in intergenic regions were excluded, whereas SNPs in genes were retained. When more than two annotated SNPs were in the same gene, the constant of linkage disequilibrium strength (Gabriel’s D’) was calculated among SNPs using the 1KJPN reference panel dataset. One SNP was selected from SNPs in strong linkage disequilibrium with the D’ values of more than 90 in the same gene. In this selection by Gabriel’s D’, SNPs in exons were preferred to SNPs in introns. One SNP in an exon was ultimately selected in each gene to yield the candidate SNPs.

### Genotyping by qPCR in the two cohorts

The final set of candidate SNPs was genotyped in all patients in both cohorts using the TaqMan SNP Genotyping Assay (Thermo Fisher Scientific Inc., Waltham, MA) according to the manufacturer’s instructions. Quantitative PCR conditions were 1 cycle of 95 °C for 10 min, and 40 cycles of 95 °C for 15 s and 60 °C for 1 min.

### In silico prediction of SNP function

Transcription factor binding sites in SNP-containing sequences were predicted with the TRANSFAC program of RegulomeDB [22]. Phenotypes associated with identified transcription factors and their encoding genes were investigated in the Human Phenotype Ontology database [23].

### Statistical analysis

Fentanyl blood concentration data were compared between the patients with and without PONV using a Mann–Whitney test. In the genome-wide association study, associations of the incidence of PONV with the alleles of each SNP were tested using a Fisher’s exact test. In the genotyping by qPCR analysis, deviation of observed genotype frequencies from HWE was tested at each SNP using a chi-square test. Associations of PONV incidence with SNP genotypes or alleles were tested using a chi-square test. We also performed a multivariate analysis combining genetic and clinical factors in a logistic regression model. Differences were considered significant at *p* < 0.05 unless otherwise noted.

A power analysis was performed to estimate the necessary sample size to achieve statistical power of greater than 0.8 when detecting a statistically significant (*p* < 0.05) genetic effect of a 15% difference in incidence of PONV between alleles using an additive genetic model. The minor allele frequency was presumptively set to 0.20. We calculated the minimum sample size as 218 patients.

## Results

The genomic DNA from eight of the 264 enrolled patients had insufficient quality for genotyping, so only data from the remaining 256 patients were analyzed. Table 1 summarizes demographic and clinical characteristics of patients in both cohorts.

**Table 1:** Characteristics of the Epi and IV-PCA cohorts

	Epi cohort	IV-PCA cohort	All
Number	174	82	256
Age (years)	67 ± 13	68 ± 12	68 ± 13
Male/female	112/62	32/50	144/112
Height (cm)	159 ± 9	153 ± 9	158 ± 9
Weight (kg)	59 ± 10	58 ± 10	59 ± 10
Smoking status (smokers/non-smokers)	35/139	10/72	45/211
History of motion sickness (yes/no)	19/155	16/66	35/221
Type of surgery			
Abdominal (no laparoscopic)	138	7	145
Abdominal (laparoscopic)	31	26	57
Spine	0	19	19
Upper extremities	0	3	3
Lower extremities	0	10	10
Mastectomy	0	11	11
Thyroidectomy	0	2	2
Unclassifiable	5	4	9
Duration of surgery (min)	192 ± 122	160 ± 104	182 ± 117
Use of nitrous oxide (used/not used)	62/112	50/32	112/144

Data are presented as mean ± S.D. or number.

Twenty-four hours after the end of surgery, the PONV incidence in the Epi and IV-PCA cohorts was 20% (35/174) and 28% (23/82), respectively. In the Epi cohort, the fentanyl blood concentration of patients with PONV was not significantly higher than that of patients without PONV [0.11 (0.24) vs. 0.04 (0.12) ng/mL, median (interquartile range), *p* = 0.22]. In the IV-PCA cohort, the fentanyl blood concentration of patients with PONV was also not significantly higher than that of patients without PONV [0.62 (0.64) vs. 0.52 (0.45) ng/mL, *p* = 0.76].

**Fig. 1:**
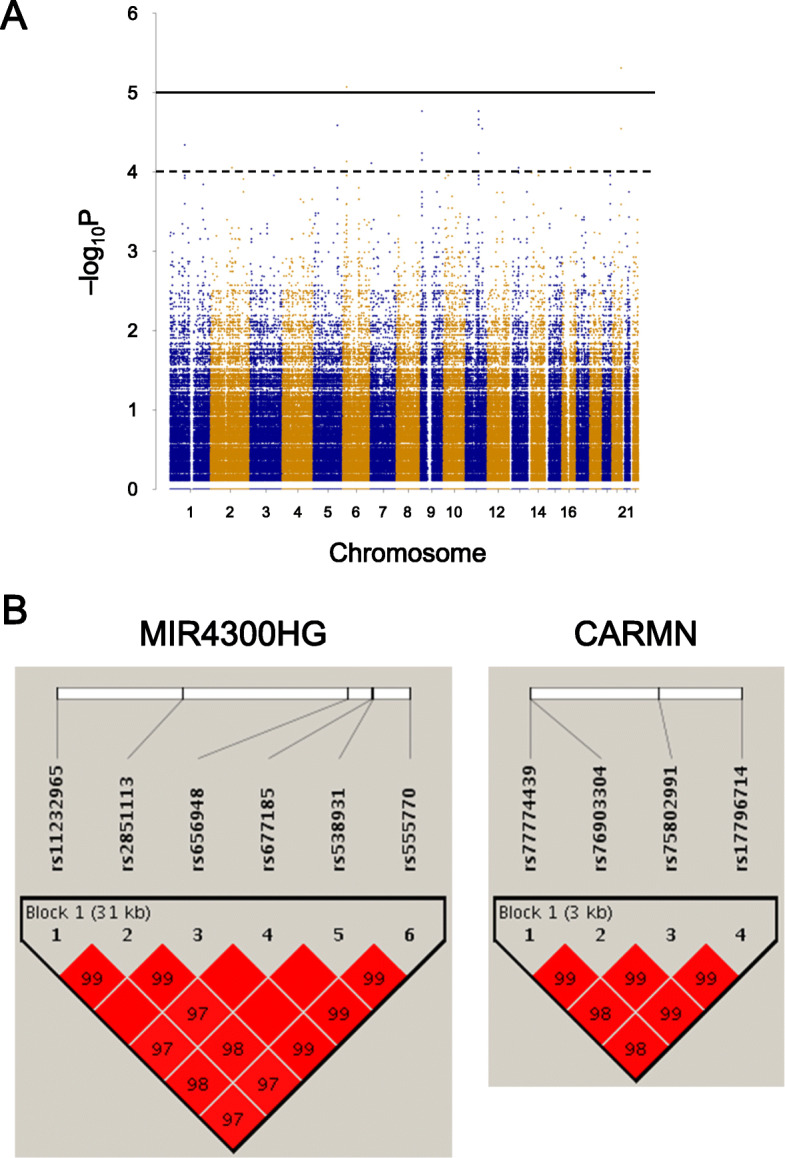
**a** A Manhattan plot of Fisher’**s** exact test *p* values against chromosomal location. The solid line represents a significance level of *p* < 10^−5^, and the dashed line represents significance at *p* < 10^−4^, **b** Pairwise linkage disequilibrium plots for SNPs in *CARMN* (right) and *MIR4300HG* (left). The plots were calculated using the Japan Reference Genome data. All SNPs were in strong linkage disequilibrium (All Gabriel’s D’ > 96)

Twenty-four patients were genotyped at the 659,636 lead SNPs via DNA microarray. The average call rate across samples was 99.4% (median 100%, 90% percentile 100%, 10% percentile 100%). Moreover, 97% of SNPs (639,754 SNPs/659,636 SNPs) on the microarray had a call rate above 95%. The complete genotypes of the remaining 24,330,529 SNPs were identified via imputation. Figure 1a shows the detailed *p* values for all SNPs after imputation. We observed 2 SNPs associated with PONV incidence when assuming statistical significance at *p* < 10^−5^ (Fig. 1a). With a significance threshold of *p* < 10^−4^, there were 78 additional high-confidence SNPs associated with PONV (Fig. 1a).

Investigation of these 78 SNPs with the Variant Annotation Integrator revealed 15 had known structural and functional annotations. Table 2 shows the annotations of each of these SNPs. Three SNPs were located in intergenic regions. Two SNPs were located in the introns of protein-coding genes (i.e., *TNRC18* and *PTPRD*). Ten SNPs were located in long non-coding RNA (lncRNA) genes (i.e., *CARMN* and *MIR4300HG*). Of the 10 SNPs in the two lncRNA genes, 4 of the SNPs in *CARMN* were in complete linkage disequilibrium (Fig. 1b). The six SNPs in *MIR4300HG* were also in complete linkage disequilibrium (Fig. 1b). We ultimately chose 4 candidate SNPs for downstream individual genotyping: rs17796714 (*CARMN*, row 5 in Table 2), rs13234328 (*TNRC18*, row 6), rs1333114 (*PTPRD*, row 7), and rs11232965 (*MIR4300HG*, row 8).

**Table 2:** Annotations of 15 SNPs associated with PONV in the initial 24 patients

rs#	Chr#	Position	Gene	Function	Annotation
rs10051593	5	7184639	intergenic	Regulatory region variant	DNHS
rs77774439	5	148796658	CARMN	Regulatory region variant	TFBS (of EP300)
rs76903304	5	148796677	CARMN	Regulatory region variant	TFBS (of EP300)
rs75802991	5	148798743	CARMN	Regulatory region variant	DNHS
rs17796714	5	148800089	CARMN	Non-coding transcript exon variant	2^nd^ exon of 6 exons
rs13234328	7	5434598	TNRC18	Regulatory region variant	DNHS
rs1333114	9	9446327	PTPRD	Regulatory region variant	TFBS (of MAFF, MAFK)
rs11232965	11	81726749	MIR4300HG	Non-coding transcript exon variant	6^th^ exon of 8 exons
rs2851113	11	81737964	MIR4300HG	Regulatory region variant	DNHS
rs656948	11	81752743	MIR4300HG	Regulatory region variant	TFBS (of GATA3)
rs677185	11	81754814	MIR4300HG	Regulatory region variant	DNHS
rs538931	11	81754983	MIR4300HG	Regulatory region variant	TFBS (of GATA3)
rs555770	11	81758202	MIR4300HG	Regulatory region variant	TFBS (of POLR2A)
rs4325818	20	56054383	intergenic	Regulatory region variant	TFBS (of POLR2A, CTCF, MYC), DNHS
rs6123702	20	56055633	intergenic	Regulatory region variant	TFBS (of POLR2A, MYC, MEF2C), DNHS

*rs#* SNP identifier at dbSNP, *Chr#* chromosome number, *Position* chromosome position in GRCh37/hg19 assembly, *DNHS* DNase-I hypersensitive site, *TFBS* transcription factor binding site

**Fig. 2:**
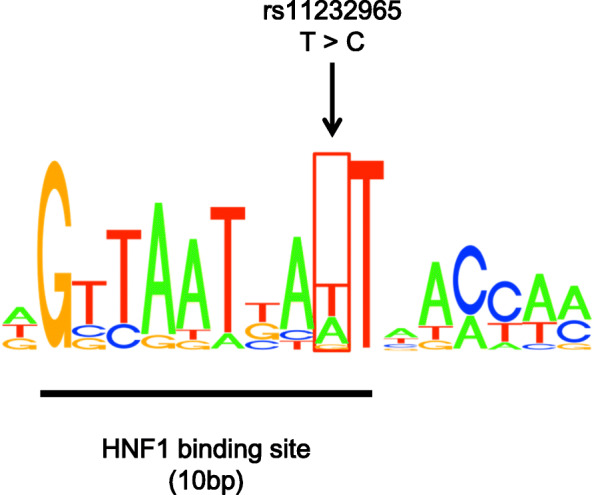
The sequence of part of the *MIR4300HG* gene (ch11: 81,726,739–81,726,756). The total height of the letters depicts the information content of sites where transcription factors bind. The relative size of each letter indicates their frequency in the sequence. An HNF1 motif was identified via in silico prediction, noted as the 10 base-sequence with taller letters. The location of the SNP rs11232965 falls within that sequence (arrow)

These four candidate SNPs were genotyped with qPCR in the 256 patients in both cohorts. The call rate was 99.6% (255/256) in the qPCR experiments. Table 3 shows the incidence of PONV for genotypes or alleles of these four candidate SNPs 24 h after the end of surgery. The variant rs1333114-SNP in *PTPRD* was associated with the incidence of PONV. Although there was no significant difference among genotypes (*p* = 0.07), the minor allele (A) at this SNP was significantly associated with a reduced incidence of PONV (*p* = 0.02). The variant rs11232965 in *MIR4300HG* was also significantly associated with reduced incidence of PONV (Table 3). There were significant differences in the incidence of PONV among genotypes and between alleles at this SNP (*p* = 0.01 and 0.007). Additionally, in silico prediction identified a 10-bp HNF1-motif within the *MIR4300HG* gene that contains rs11232965-SNP (Fig. 2). *HNF1A* and *HNF1B*, which encode the HNF1 transcription factor, are involved in 15 and 113 known phenotypes in humans (e.g., pica behavior), respectively (Accessed to Human Phenotype Ontology database on March 4, 2017).

**Table 3:** Association of each candidate SNP with incidence of PONV in all 255 patients

rs#	Gene	Variant	MAF	HWE	Genotypes			*P*	Alleles		*P*
					Wild-type (case/con)	Hetero (case/con)	Homo (case/con)		Major (case/con)	Minor (case/con)	
rs17796714	CARMN	G > A	0.31	0.86	25/97	22/86	10/15	0.08	72/282	42/116	0.12
rs13234328	TNRC18	G > A	0.23	0.36	37/112	16/79	4/7	0.18	90/303	24/93	0.59
rs1333114	PTPRD	G > A	0.37	0.92	29/72	24/94	4/32	0.07	82/238	32/158	0.02
rs11232965	MIR4300HG	T > C	0.42	0.44	29/58	20/99	8/41	0.01	78/215	36/181	0.007

Incidences are presented as number

*rs#* SNP identifier at dbSNP, *MAF* minor allele frequency, *HWE p* value of deviance from Hardy–Weinberg equilibrium, *Hetero* heterozygous mutant, *Homo* homozygous mutant, *Major* major allele, *Minor* minor allele, *case* patients with PONV, *con* patients without PONV, *P p* values in statistics

To estimate the impact of clinical and genetic factors on PONV incidence, we used a multivariate analysis (i.e., logistic regression analysis). Female sex, history of motion sickness, and carrying a C allele at rs11232965-SNP were identified as independent PONV predictors (Table 4). The odds ratio of carrying a C allele at rs11232965-SNP was 0.16-fold between the patients with and without PONV incidence.

**Table 4:** Risk factors for incidence of PONV in multivariate analysis

Variable	Odds ratio	95% CI	*p* value
Female sex	14.7	4.16–51.9	0.00003
Non-smoker	0.55	0.09–3.42	0.52
History of motion sickness	3.89	1.02–14.8	0.046
Use of nitrous oxide	0.78	0.24–2.55	0.26
Duration of surgery	1.00	0.99–1.00	0.59
Fentanyl concentration	1.64	0.31–8.83	0.57
rs1333114 G > A	0.48	0.16–1.44	0.19
rs11232965 T > C	0.16	0.05–0.51	0.002

*CI* Confidence interval

## Discussion

To identify SNPs for PONV, we first identified 78 SNPs associated with PONV incidence using a DNA microarray optimized for genotyping in Japanese populations. Variants at these 78 SNPs were associated with the PONV incidence in a limited subpopulation (i.e., female patients who underwent mastectomy or gynecological surgery). Secondly, we focused on four of these 78 SNPs identified as candidates by in silico structural and functional annotation according to previously described methods [24, 25]. We finally verified the potential association of two of these four candidate SNPs with the incidence of PONV in a more general population of both male and female patients that underwent different surgical procedures.

The most statistically significant SNP was rs11232965-SNP in *MIR4300HG* (Table 3). This variant was also a strong predictor of PONV protection (Table 4). Although this gene encodes the long non-coding RNA (lncRNA) MIR4300HG, its biological function remains unknown [26]. No phenotypes associated with variable genotypes at this SNP have been reported. Although more than 10,000 lncRNAs have been identified in the human genome [27, 28], the biological function and significance of the majority of them has yet to be elucidated. Some lncRNAs play a role in transcriptional, post-transcriptional, and epigenetic regulation in cells [29]. In the central nervous system, lncRNAs may modulate stress responses and mediate aspects of brain evolution, development, homeostasis, and plasticity [29, 30]. If the lncRNA MIR4300HG is involved in PONV development, genetic variants of *MIR4300HG* may offer protection from PONV. Interestingly, a few studies have previously suggested that genetic variation in lncRNA genes can directly cause disease and influence disease susceptibility [31, 32]. SNPs occurring in lncRNA genes may affect their function and result in individual differences in gene expression and phenotype [33]. These findings offer one interpretation for our observations. Moreover, we found that rs11232965s falls in a HNF1 transcription factor binding site (Fig. 2). This DNA-binding protein may bind to this region. The genes that encode it (i.e., HNF1A and HNF1B) are associated with several known phenotypes. Interestingly, one phenotype associated with *HNF1B* is pica behavior, the persistent intake of non-food substances. Pica of kaolin clay in rodents has been used as a research alternative to vomiting behavior [34]. This may explain how changes in *MIR4300HG* expression regulated by the HNF1 transcription factor could influence the molecular and cellular mechanisms of PONV.

The variant rs1333114-SNP in *PTPRD* also protects against PONV (Table 3). This gene encodes the protein tyrosine phosphatase receptor type delta and regulates long-term potentiation of synaptic transmission in the hippocampus [35]. Previous reports suggest that PTPRD deficiency in mice is semilethal due to insufficient food intake behaviors [35]. Our results may simply show a decreased appetite following nausea and/or vomiting alters *PTPRD* expression in the brain. Nevertheless, rs1333114-SNP variation was not a statistically significant genetic factor in the multiple logistic regression analysis (Table 4). The effect magnitude of variation at rs1333114-SNP may be small. We may need to analyze the genotypes of all 255 patients to calculate statistical *p* values. The *p* values of four independent association tests with four candidate SNPs should be adjusted with a Bonferroni correction for multiple comparison (i.e., statistical *α* value 0.05/4 for detection of true-positive difference). Accordingly, differences in PONV incidence between alleles of rs1333114-SNP in *PTPRD* may only be called a trend. Only rs11232965 in *MIR4300HG* was significantly associated with PONV incidence according to stringent statistical criteria.

Furthermore, the multiple logistic regression analysis indicated a strong association between rs11232965-SNP in *MIR4300HG* and female sex with PONV incidence (Table 4). Female sex per se was previously identified as the strongest PONV risk factor [2], and our current and previous results [8] confirm this (the odds ratios were 14.7-fold and 7.15-fold, respectively). Sex is a genetic trait, and we speculate sex differences in PONV may involve variable regulation by lncRNAs or transcription factors. Indeed, our previous study showed polymorphism in *TACR1* located where estrogen may act as a transcription factor was associated with PONV incidence and severity [8]. In addition, a recent study suggested that the lncRNA *MIR4300HG* and the microRNA *miR-4300* may be related to estrogen receptor function [36]. In this interesting report, in silico analyses identified 55 genes interacting with *miR-4300*. Interestingly, the *ESR1* gene, which encodes the estrogen receptor, was also identified among these 55 genes. Thus, the lncRNA *MIR4300HG* may regulate the development of PONV via the estrogen signaling pathway.

Contrary to expectation, non-smoking status, history of motion sickness, nitrous oxide use, and surgery duration were not identified as risk factors in this study. This observation is consistent with results of our previous study in a different Japanese cohort [8]. Surprisingly, fentanyl concentration was also not a PONV risk factor. As described in the results, there were no significant differences in blood fentanyl concentration between patients with and without PONV in either cohort. In the Epi cohort, PONV was initiated at a lower fentanyl concentration than the concentration needed for analgesia (> 1 ng/mL). Ginosar and colleagues showed that in epidural fentanyl infusion PONV incidence was not dose-dependent [37]. Likewise, in the IV-PCA cohort, the PONV incidence after intravenous administration of fentanyl was not related to the fentanyl concentration. We can only speculate that individual differences in the pharmacodynamic profile of fentanyl are greater than differences in pharmacokinetic profiles.

One limitation of this study is our choice of 1 × 10^−4^ as the significance threshold. Studies with a genome-wide design commonly adopted 1 × 10^−7^ as the significance threshold because modern genotyping technologies can capture hundreds of thousands of SNPs and ~ 500,000 independent statistical tests are simultaneously performed (i.e., statistical *α* value of 0.05/500,000 is required to detect true differences) [38]. Such high-powered genome-wide association studies are expensive. To reduce costs, we reduced the number of samples below the convention regardless of insufficient statistical power. To compensate, we compared allele frequencies between cases and controls in a small population (i.e., same sex and surgical procedure) and confirmed associations of candidate SNPs in a larger population. This cheaper stepwise approach is frequently used to verify and replicate genome-wide association studies [38–40].

Another limitation is that our findings may be useful for the prediction of susceptibility to PONV in specific populations. The MAFs of the SNP rs11232965 differ among different populations. The 1000 Genome database shows that the SNP rs11232965 variant is rare in the African population (MAF: 0.0023) but common in East Asians, including Japanese (MAF: 0.4058; visited on June 1, 2020; https://www.ncbi.nlm.nih.gov/snp/rs11232965#frequency_tab). The protective effect of this SNP against PONV may be limited in the Japanese population. However, differences in genetic effects among populations remain unknown.

Our results indicated that specific SNP genotypes in the *MIR4300HG* gene may be involved in susceptibility to PONV in Japanese patients. The incidence of PONV may be predicted partially using preoperative genotyping of the rs11232965-SNP in the *MIR4300HG* gene. As shown in Table 4, our current findings demonstrated that a small odds ratio was associated with the rs11232965-SNP variant. Unresolved PONV can lead to increased recovery room time, expanded nursing care, and potential hospital admission—all factors that may increase total health care costs. Equally important are high levels of patient discomfort and dissatisfaction associated with PONV.

## Conclusions

We identified a novel SNP (rs11232965) associated with PONV located in the lncRNA *MIR4300HG*. The rs11232965-SNP variant (T > C) protects against PONV incidence and severity. This suggests potential molecular mechanisms for PONV and may allow PONV prediction.

## Data Availability

The raw datasets of DNA microarray during the current study are available from the corresponding author on reasonable request. The authors will facilitate access to the raw data at the Japanese Genotype-phenotype Archive (JGA, https://www.ddbj.nig.ac.jp/jga/index-e.html).

## Abbreviations

PONV: Postoperative nausea and vomiting
SNPs: Single-nucleotide polymorphisms
lncRNA: Long non-coding RNA
IV-PCA: Intravenous patient-controlled analgesia
HWE: Hardy–Weinberg equilibrium
MAF: Minor allele frequency

## References

[1] Gan TJ, Diemunsch P, Habib AS, Kovac A, Kranke P, Meyer TA (2014). Consensus guidelines for the management of postoperative nausea and vomiting. Anesth Analg..

[2] Apfel CC, Korttila K, Abdalla M, Kerger H, Turan A, Vedder I (2004). A factorial trial of six interventions for the prevention of postoperative nausea and vomiting. N Engl J Med..

[3] Janicki PK, Sugino S (2014). Genetic factors associated with pharmacotherapy and background sensitivity to postoperative and chemotherapy-induced nausea and vomiting. Exp Brain Res..

[4] Landau R, Janicki PK (2018). Risk-tailored prophylaxis for postoperative nausea and vomiting: still a messy issue. Br J Anaesth..

[5] Sugino S, Hayase T, Higuchi M, Saito K, Moriya H, Kumeta Y (2014). Association of μ-opioid receptor gene (OPRM1) haplotypes with postoperative nausea and vomiting. Exp Brain Res..

[6] Walter C, Lötsch J (2009). Meta-analysis of the relevance of the OPRM1 118A>G genetic variant for pain treatment. Pain..

[7] Rueffert H, Thieme V, Wallenborn J, Lemnitz N, Bergmann A, Rudlof K (2009). Do variations in the 5-HT3A and 5-HT3B serotonin receptor genes (HTR3A and HTR3B) influence the occurrence of postoperative vomiting?. Anesth Analg..

[8] Hayase T, Sugino S, Moriya H, Yamakage M (2015). TACR1 gene polymorphism and sex differences in postoperative nausea and vomiting. Anaesthesia..

[9] Janicki PK, Vealey R, Liu J, Escajeda J, Postula M, Welker K (2011). Genome-wide Association study using pooled DNA to identify candidate markers mediating susceptibility to postoperative nausea and vomiting. Anesthesiology..

[10] Klenke S, de Vries GJ, Schiefer L, Seyffert N, Bachmann HS, Peters J (2018). CHRM3 rs2165870 polymorphism is independently associated with postoperative nausea and vomiting, but combined prophylaxis is effective. Br J Anaesth..

[11] Tan E, Lim ECP, Teo Y, Lim Y, Law H, Sia AT (2009). Ethnicity and OPRM variant independently predict pain perception and patient-controlled analgesia usage for post-operative pain. Mol Pain..

[12] Kasai S, Ikeda K (2011). Pharmacogenomics of the human μ-opioid receptor. Pharmacogenomics..

[13] Kuriyama S, Yaegashi N, Nagami F, Arai T, Kawaguchi Y, Osumi N (2016). The Tohoku Medical Megabank Project: design and mission. J Epidemiol..

[14] Nagasaki M, Yasuda J, Katsuoka F, Nariai N, Kojima K, Kawai Y (2015). Rare variant discovery by deep whole-genome sequencing of 1,070 Japanese individuals. Nat Commun..

[15] Kawai Y, Mimori T, Kojima K, Nariai N, Danjoh I, Saito R (2015). Japonica array: improved genotype imputation by designing a population-specific SNP array with 1070 Japanese individuals. J Hum Genet..

[16] Yamaguchi-Kabata Y, Nariai N, Kawai Y, Sato Y, Kojima K, Tateno M (2015). iJGVD: an integrative Japanese genome variation database based on whole-genome sequencing. Hum Genome Var..

[17] Little J, Higgins JPT, Ioannidis JPA, Moher D, Gagnon F, von Elm E (2009). Strengthening the reporting of genetic association studies (STREGA): an extension of the STROBE statement. Eur J Epidemiol..

[18] Howie BN, Donnelly P, Marchini J (2009). A flexible and accurate genotype imputation method for the next generation of genome-wide association studies. PLoS Genet..

[19] Delaneau O, Marchini J, Zagury JF (2011). A linear complexity phasing method for thousands of genomes. Nat Methods..

[20] Purcell S, Neale B, Todd-Brown K, Thomas L, Ferreira MAR, Bender D (2007). PLINK: a tool set for whole-genome association and population-based linkage analyses. Am J Hum Genet..

[21] Hinrichs AS, Raney BJ, Speir ML, Rhead B, Casper J, Karolchik D (2016). UCSC Data Integrator and Variant Annotation Integrator. Bioinformatics..

[22] Boyle AP, Hong EL, Hariharan M, Cheng Y, Schaub MA, Kasowski M (2012). Annotation of functional variation in personal genomes using RegulomeDB. Genome Res..

[23] Köhler S, Vasilevsky NA, Engelstad M, Foster E, McMurry J, Aymé S (2017). The human phenotype ontology in 2017. Nucleic Acids Res..

[24] Nishizaki SS, Boyle AP (2017). Mining the unknown: assigning function to noncoding single nucleotide polymorphisms. Trends Genet..

[25] Edwards SL, Beesley J, French JD, Dunning M (2013). Beyond GWASs: Illuminating the dark road from association to function. Am J Hum Genet..

[26] Strausberg RL, Feingold EA, Grouse LH, Derge JG, Klausner RD, Collins FS (2002). Generation and initial analysis of more than 15,000 full-length human and mouse cDNA sequences. Proc Natl Acad Sci U S A..

[27] Ulitsky I, Bartel DP (2013). lincRNAs: genomics, evolution, and mechanisms. Cell..

[28] Hon CC, Ramilowski JA, Harshbarger J, Bertin N, Rackham OJL, Gough J (2017). An atlas of human long non-coding RNAs with accurate 5’ ends. Nature..

[29] Qureshi IA, Mehler MF (2013). Long non-coding RNAs: novel targets for nervous system disease diagnosis and therapy. Neurotherapeutics..

[30] Qureshi IA, Mehler MF (2012). Emerging roles of non-coding RNAs in brain evolution, development, plasticity and disease. Nat Rev Neurosci..

[31] Cartault F, Munier P, Benko E, Desguerre I, Hanein S, Boddaert N (2012). Mutation in a primate-conserved retrotransposon reveals a noncoding RNA as a mediator of infantile encephalopathy. Proc Natl Acad Sci U S A..

[32] Ning S, Wang P, Ye J, Li X, Li R, Zhao Z (2013). A global map for dissecting phenotypic variants in human lincRNAs. Eur J Hum Genet..

[33] Chen G, Qiu C, Zhang Q, Liu B, Cui Q (2013). Genome-wide analysis of human SNPs at long intergenic noncoding RNAs. Hum Mutat..

[34] Horn CC (2008). Why is the neurobiology of nausea and vomiting so important?. Appetite..

[35] Uetani N, Kato K, Ogura H, Mizuno K, Kawano K, Mikoshiba K (2000). Impaired learning with enhanced hippocampal long-term potentiation in PTPdelta-deficient mice. EMBO J..

[36] Ogura Y, Kou I, Takahashi Y, Takeda K, Minami S, Kawakami N (2017). A functional variant in MIR4300HG, the host gene of microRNA MIR4300 is associated with progression of adolescent idiopathic scoliosis. Hum Mol Genet..

[37] Ginosar Y, Riley ET, Angst MS (2003). The site of action of epidural fentanyl in humans: the difference between infusion and bolus administration. Anesth Analg..

[38] McCarthy MI, Abecasis GR, Cardon LR, Goldstein DB, Little J, Ioannidis JPA (2008). Genome-wide association studies for complex traits: consensus, uncertainty and challenges. Nat Rev Genet..

[39] Saito A, Kamatani N (2002). Strategies for genome-wide association studies: optimization of study designs by the stepwise focusing method. J Hum Genet..

[40] Skol AD, Scott LJ, Abecasis GR, Boehnke M (2006). Joint analysis is more efficient than replication-based analysis for two-stage genome-wide association studies. Nat Genet..

